# Source Apportionment and Risk Assessment of Emerging Contaminants: An Approach of Pharmaco-Signature in Water Systems

**DOI:** 10.1371/journal.pone.0122813

**Published:** 2015-04-15

**Authors:** Jheng Jie Jiang, Chon Lin Lee, Meng Der Fang, Kenneth G. Boyd, Stuart W. Gibb

**Affiliations:** 1 Department of Marine Environment and Engineering, National Sun Yat-sen University, Kaohsiung, Taiwan; 2 Department of Public Health, College of Health Science, Kaohsiung Medical University, Kaohsiung, Taiwan; 3 Asia-Pacific Ocean Research Center, National Sun Yat-sen University, Kaohsiung, Taiwan; 4 Research Center of Environmental Medicine, Kaohsiung Medical University, Kaohsiung, Taiwan; 5 Green Energy and Environment Research Laboratories, Industrial Technology Research Institute, Hsinchu, Taiwan; 6 Environmental Research Institute (ERI), North Highland College, University of the Highlands and Islands, Thurso, Caithness, Scotland, United Kingdom; Purdue University, UNITED STATES

## Abstract

This paper presents a methodology based on multivariate data analysis for characterizing potential source contributions of emerging contaminants (ECs) detected in 26 river water samples across multi-scape regions during dry and wet seasons. Based on this methodology, we unveil an approach toward potential source contributions of ECs, a concept we refer to as the “Pharmaco-signature.” Exploratory analysis of data points has been carried out by unsupervised pattern recognition (hierarchical cluster analysis, HCA) and receptor model (principal component analysis-multiple linear regression, PCA-MLR) in an attempt to demonstrate significant source contributions of ECs in different land-use zone. Robust cluster solutions grouped the database according to different EC profiles. PCA-MLR identified that 58.9% of the mean summed ECs were contributed by domestic impact, 9.7% by antibiotics application, and 31.4% by drug abuse. Diclofenac, ibuprofen, codeine, ampicillin, tetracycline, and erythromycin-H2O have significant pollution risk quotients (RQ>1), indicating potentially high risk to aquatic organisms in Taiwan.

## Introduction

Emerging contaminants (ECs) are mainly substances that many of them are unregulated or inadequately regulated and has raised the public attention to their presence in the environment used by different kinds of aspect, for instance, industrial and domestic [1,2]. The occurrence and fate of ECs in aquatic environments have been widely studied. Increasing contamination of aquatic systems by ECs is a major problem for aquatic life, as well as for human health, as they are highly mobile and often of toxicological concern [3–6]. Pharmaceuticals and personal care products (PPCPs), as well as illicit drugs, are increasingly discharged with wastewater to surface water environments [7–9]. Several direct and indirect pathways are available for introduction of ECs into an aqueous environment. One primary route is via effluent from municipal wastewater treatment plants (WWTPs) [10–12]. Since wastewater treatment processes are designed primarily to remove pathogens, suspended particles, and nutrients from sewage, removal of ECs is purely incidental and their elimination varies [10,13]. Several authors have documented conventional wastewater treatment showed inadequate on ECs removal [11,14,15]. Several ECs may be susceptible to degradation or transformation, but their continuous introduction into the aquatic environment in reality confers some degree of pseudo-persistence [10,16]. Although these compounds occur at relatively low concentrations, their continual long-term release may nevertheless result in significant environmental impacts.

According to statistical data from the Taiwan Food and Drug Administration, drug disposal in Taiwan amounts to 36 tons per year, and total medical expenses in 2011 reached 48 billion US dollars [17]. Therefore, the large amounts of unconsumed drugs may be present in the water systems. Available information concerning ECs in Taiwan is still limited. Few recent studies focus on selected sampling locations (industrial and hospital) for certain pharmaceuticals in northern Taiwan [18,19], while the occurrence of ECs in the water systems of southern Taiwan, particularly any effect on water quality in adjacent areas, remains unknown.

Multivariate statistical techniques, such as receptor model and cluster analysis, have been widely used to apportion the contributions of contaminants derived from different sources and investigate the distribution pattern and association of contaminants in the environment [20,21]. In addition, taking into account the ubiquity of the selected ECs, the relative abundance of contaminants, as opposed to absolute concentrations, can be considered as a chemical signature specific to a source contribution or contaminant plume. This chemical signature can help to better understand the fate and contribution of ECs in aquatic environments.

This study develops a methodology for a concept we refer to as the “Pharmaco-signature” for a source assessment of ECs in the particular land-use zone with a particular contribution of a mixture of ECs. The methodology is built upon a comprehensive and exploratory multivariate data analysis including the principal component analysis-multiple linear regression model (PCA-MLR) and the hierarchical cluster analysis (HCA). This methodology makes it possible to (a) obtain more information about the structure of the data; and (b) separate and discern the source contributions of ECs. Results of this study could provide information on levels, sources and potential risks of ECs, and for protecting water resources and environmental management in Taiwan.

## Materials and Methods

### Ethics Statement

For sampling in the four rivers of Kaohsiung, no specific permit was required for the described field study. The study location is not privately owned or protected in any way and we confirm that the field study did not involve endangered or protected species.

### Materials

The chemicals and standards used (including suppliers, purities, and detailed physicochemical properties of the 28 selected ECs) are described in S1 Text and S1 Table of the Supporting Information.

### Study area and sample collection

**Fig 1:**
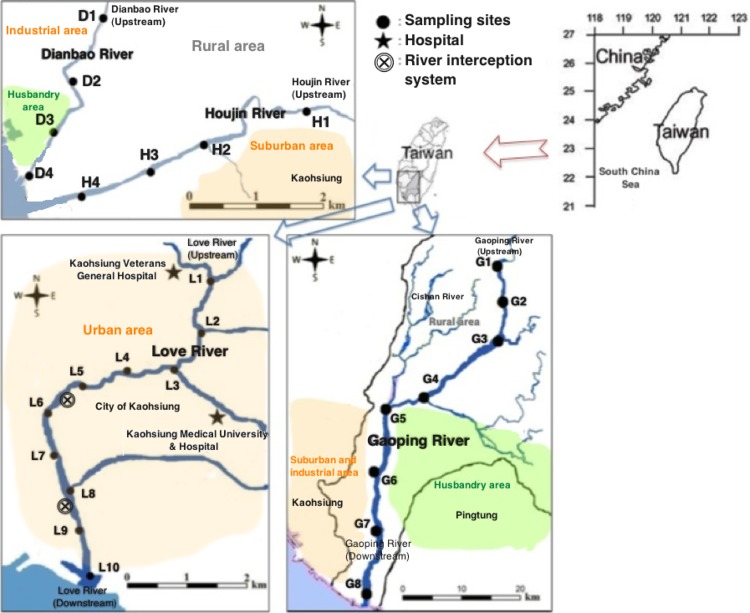
Location of the water systems and sampling points in southern Taiwan. Sites G1-G8 are located on the Gaoping River. Sites L1-L10 are on the Love River. Sites H1-H4 are on the Houjin River and sites D1-D4 are on the Dianbao River.

The study area covers the entirety of the urban, suburban, animal husbandry, and rural districts of Kaohsiung (22°18’ N, 120°38’ E), which has a population of 3 million and is also the largest industrial city in Taiwan. A map of the four selected rivers and our sampling locations are shown in Fig 1. Detailed description and coordinates of the sampling sites is included in the Table 1. Like many other rivers in Taiwan, these four rivers receive a variety of wastewaters from untreated domestic wastewater and/or animal husbandry discharge [22]. Gaoping River has the largest drainage basin, including rural, suburban, animal husbandry, and industrial regions of Kaohsiung, with an area of 3,256 km^2^. Gaoping River is also the longest river in Taiwan, with a length of approximately 140 km. Love River flows through the most urbanized and densely populated area of Kaohsiung City, with a length of 16.4 km and a 56 km^2^ drainage area. Houjin River and Dianbao River have drainage basins of 70.4 and 107.1 km^2^ and lengths of 21 and 25 km, respectively. Both rivers drain a partially rural region, with one tributary (located near H2) of the Houjin River flows through a suburban area, and downstream Dianbao River flows through an animal husbandry area. Two sampling campaigns were conducted in April 2010 (dry season) and July 2013 (wet season) at the water systems, with sampling sites denoted as follows: Gaoping River (sites G1-G8), Love River (L1-L10), Houjin River (H1-H4), and Dianbao River (D1-D4). Surface water samples (1L) in duplicate were collected in pre-cleaned amber glass bottles at each sampling site. All of the samples were stored in a cooler during sampling campaigns and were immediately transported to the laboratory.

**Table 1:** Detailed description and coordinates of the sampling sites in the water systems.

Site	Latitude	Longitude	Type	Influence	Note
**Gaoping River**					
G1	N 23.047°	E 120.668°	Fresh water	Rural	
G2	N 22.995°	E 120.638°	Fresh water	Rural	
G3	N 22.885°	E 120.640°	Fresh water	Rural	
G4	N 22.798°	E 120.512°	Fresh water	Rural	
G5	N 22.770°	E 120.451°	Fresh water	Husbandry	
G6	N 22.646°	E 120.437°	Fresh water	Husbandry/Industrial/Urban	
G7	N 22.593°	E 120.440°	Fresh water	Husbandry/Industrial/Urban	
G8	N 22.498°	E 120.420°	Brackish water	Industrial	
**Love River**					
L1	N 22.677°	E 120.322°	Fresh water	Urban	Kaohsiung Veterans General Hospital
L2	N 22.659°	E 120.311°	Fresh water	Urban	Tributary
L3	N 22.653°	E 120.306°	Fresh water	Urban	Kaohsiung Medical University & Hospital
L4	N 22.652°	E 120.296°	Fresh water	Urban	
L5	N 22.650°	E 120.288°	Fresh water	Urban	
L6	N 22.645°	E 120.281°	Fresh water	Urban	River interception station
L7	N 22.640°	E 120.283°	Fresh water	Urban	
L8	N 22.632°	E 120.286°	Fresh water	Urban	
L9	N 22.626°	E 120.288°	Fresh water	Urban	River interception station
L10	N 22.620°	E 120.290°	Brackish water	Urban	
**Houjin River**					
H1	N 22.729°	E 120.314°	Fresh water	Rural/Suburban	
H2	N 22.724°	E 120.291°	Fresh water	Suburban	
H3	N 22.720°	E 120.281°	Fresh water	Suburban	
H4	N 22.714°	E 120.261°	Brackish water	Suburban	
**Dianbao River**					
D1	N 22.752°	E 120.273°	Fresh water	Rural/Industrial	
D2	N 22.734°	E 120.264°	Fresh water	Industrial	
D3	N 22.726°	E 120.262°	Fresh water	Husbandry	
D4	N 22.718°	E 120.255°	Brackish water	Husbandry	

### Sample preparation and analysis

Chemical analysis of ECs followed the methods employed in our previous study [23]. Water samples were filtered through 0.7 μm glass fiber filters, then acidified to pH = 6 by adding 0.1 M HCl, followed by addition of 0.2 g/L Na_2_EDTA as the chelating agent. For solid-phase extraction (SPE) of water samples, 300 mL water samples were spiked with acetaminophen-d_4_, amphetamine-d_11_, methamphetamine-d_14_, MDMA-d_5_, ^13^C_6_-ibuprofen, and ^13^C_3_-caffeine as isotopically labelled surrogates in quantifying procedural recovery. An Oasis HLB cartridge (500 mg, 6 mL, Waters, Milfort, USA) was conditioned with 6 mL methanol and 6 mL deionized water. The water sample was then passed through the pre-conditioned SPE-cartridge at a flow rate of approximately 20 mL/min. Then, the cartridge was rinsed with 6 mL deionized (DI) water and dried for 30 min using the vacuum of the SPE manifold. The analyte was then eluted by 6 mL of methanol. The extract was evaporated to dryness under a gentle nitrogen stream. Afterwards, the residue was re-dissolved in a final 1 mL volume with a 50:50 (v/v) solution of methanol in DI water and filtered through a 0.22 μm filter and analyzed by liquid chromatography-tandem mass spectrometry coupled with electrospray ionization (LC-ESI-MS/MS).

Chromatography was performed using an Agilent 1200 module (Agilent Technologies, Palo Alto, CA, USA). The injection volume for PPCPs and illicit drugs was 50 and 10 μL, respectively, and the auto-sampler was operated at room temperature. Separation of PPCPs was performed on a 150 × 4.6 mm ZORBAX Eclipse XDB-C18 column with a 5 μm particle size (Agilent, Palo Alto, CA, USA). Illicit drugs were separated on a Kinetex PFP column (Phenomenex, Torrance, CA, USA, 100 × 2.1 mm, 2.6 μm). The gradients and mass spectrometer conditions used are described in the S1 Text.

### Method validation and quality control

For all the compounds, wide linearity ranges were obtained for the quantification. Seven to ten points’ calibration curves were constructed using least-squares linear regression analysis, and subjecting them to the same SPE procedures used for the environmental water samples (river waters) spiked with the analytes, typically from 0.5 to 2000 ng/L with *r* ^2^ > 0.9991 for all compounds. Recovery experiments were performed on DI water and river water samples spiked with 500 ng/L target analytes and isotopically labelled surrogates to estimate the precision, recovery, and accuracy of the analytical method. Table 2 presents the recoveries for the target analytes in DI water and river water. Mean recoveries in DI water range from 74 to 110%, and in river water they range from 76 to 115%. Mean recoveries of the isotopically labelled surrogate standards (acetaminophen-d_4_, amphetamine-d_11_, methamphetamine-d_14_, MDMA-d_5_, ^13^C_6_-ibuprofen and ^13^C_3_-caffeine) are 87 ± 11%, 74 ± 13%, 82 ± 15%, 84 ± 9%, 89 ± 8%, and 93 ± 12%, respectively. Blank samples and duplicate samples are analyzed in each batch to assure quality of the analysis. Analysis of these blanks demonstrated that the extraction and sampling procedures were free of contamination. The relative percentage difference for individual target congeners identified in paired duplicates is less than 10%. The limits of detection (LODs) are defined as three times the standard deviation of the blank samples, and the limits of quantification (LOQs) for the analytes are defined as three times the LODs (International Organization for Standardization, ISO/TS 13530, 2009). The target compound LODs ranged from 0.15 to 1.79 ng/L, and the LOQs ranged from 0.45 to 5.36 ng/L (Table 2). Overall, the validation data, such as repeatability, recoveries, and limits of detection are good, and therefore a reliable determination of the target compounds is feasible.

**Table 2:** The 28 EC compounds, their MRM pairs, recoveries in deionized (DI) water and river water, and limits of quantification (LOQ).

Chemical	DF (%)	LOQ (ng/L)	MRM1 (quantification)	MRM2 (confirmation)	Recovery (%) ± SD (n = 3)	
					DI water	River water
**NSAIDs**						
Acetaminophen	39.3	3.35	152/110	152/93	111 ± 11	115.2 ± 7.4
Diclofenac	82.1	2.80	294/250	294/214	101.3 ± 6.6	112.4 ± 9.7
Ibuprofen	100	2.53	205/161	205/158	87.7 ± 6.5	95.6 ± 7.3
Ketoprofen	89.3	5.36	252/209	-	89.8 ± 9.7	87.8 ± 8.3
Naproxen	89.3	1.72	228/169	228/184	98.6 ± 6.1	101.2 ± 3.5
Salicylic acid	85.7	2.50	136/65	136/93	99.2 ± 9.3	105.2 ± 6.4
Codeine	85.7	0.96	300/153	300/215	103.4 ± 7.3	104 ± 2.7
**Antibiotics**						
Sulfamethoxazole	85.7	0.53	254/156	254/92	105.7 ± 6.2	103.8 ± 7.0
Ampicillin	75	4.05	350/160	350/333	93.9 ± 3.9	107.3 ± 16.2
Tetracycline	92.9	5.04	445/154	445/410	86.2 ± 7.6	97.3 ± 6.8
Erythromycin-H_2_O	82.1	1.07	734/576	734/158	91.5 ± 4.2	96.8 ± 4.3
**Lipid regulator**						
Clofibric acid	39.3	5.32	213/126	213/91	86.0 ± 3.7	92.0 ± 3.1
Gemfibrozil	78.6	0.51	248/121	248/126	94.0 ± 8.3	76.1 ± 8.6
**Antiepileptic drugs**						
Carbamazepine	82.1	2.15	237/194	237/179	80.8 ± 9.4	99.3 ± 8.6
**Psychostimulants**						
Caffeine	78.6	0.75	195/138	195/110	87.4 ± 5.3	91.2 ± 7.1
**Ulcer healing**						
Omeprazole	0	1.02	346/197	346/179	74.1 ± 1.6	73.1 ± 6.5
**Sunscreen agents**						
Benzophenone-3	42.9	5.63	226/211	-	92.5 ± 8.7	92.5 ± 3.7
Benzophenone-4	75.0	1.90	306/291	306/211	103.0 ± 5.5	100.5 ± 2.4
**Illicit drugs**						
Amphetamine	42.9	1.76	136/119	136/91	101.0 ± 9.3	105.3 ± 6.7
Methamphetamine	28.6	1.28	150/119	150/91	109.6 ± 4.1	106.3 ± 3.3
Cocaine	0	1.25	304/182	304/82	103.4 ± 4.5	104.2 ± 2.2
Heroin	0	1.41	370/268	370/210	102.8 ± 6.4	109.4 ± 5.0
Ketamine	85.7	2.50	238/219	238/125	105.3 ± 4.1	97.6 ± 7.5
Pseudoephedrine	100	0.45	166/148	166/133	91.5 ± 4.0	97.7 ± 3.5
Cannabinol	0	0.61	309/279	309/171	96.7 ± 8.3	97.4 ± 5.7
Flunitrazepam	0	0.81	314/267	314/239	102.5 ± 8.5	103.8 ± 5.3
3,4-Methylenedioxymethamphetamine (MDMA)	0	0.52	194/163	194/104	102.1 ± 5.2	107.3 ± 6.5
Gamma-Hydroxybutyric acid (GHB)	32.0	2.03	103/85	103/57	97.2 ± 6.0	109.0 ± 7.2

NSAIDs: non-steroidal anti-inflammatory drugs.

DF (%): Detection frequency.

### Environmental risk assessment

Levels of environmental risk from these ECs are evaluated based on methods described by several authors [24–27]. Risk quotients (RQs) for aquatic organisms were calculated from the measured environmental concentration (MEC), and the predicted no effect concentration (PNEC) of the EC compounds. In this study, the highest concentration measured in the river waters was used for maximum MEC to calculate the maximum RQs. PNEC is calculated by dividing the lowest chronic no observed effect concentration (NOEC) by the assessment factor according to the European Technical Guidance Document [28]. A commonly used risk ranking criteria was applied: RQs <0.1 means minimal risk, 0.1≤ RQs <1 means median risk, and RQs ≥1 means high risk [29].

### Multivariate statistical analysis

Hierarchical cluster analysis (HCA) is a statistical method to classify samples into clusters through their similarity and different cluster rules. In this work, the HCA was implemented in SPSS 16.0, using Ward’s Hierarchical agglomerative method of clustering and Euclidean distance measure, to analyze the relationships among the chemical compounds. Source contribution analysis was conducted using principal component analysis-multiple linear regression (PCA-MLR) model. The purpose of PCA is to represent the total variability of the original EC data in a minimum number of factors. Each factor is orthogonal to all others, which results in the smallest possible covariance. The first factor represents the weighted (factor loadings) linear combination of the original variables (i.e., individual ECs) that account for the greatest variability. Each subsequent factor accounts for less variability than the previous factor. By critically evaluating the factor loadings, an estimate of the chemical source responsible for each factor can be made. The concentrations were Kaiser normalized and Varimax rotation was used as the preferred transformation. Multiple linear regression was than performed on the significant factors to determine the mass apportionment of each source to total concentrations. Stepwise modeling was used to allow each independent factor to enter into the regression equation if it could significantly increase the correlation, and a default significant level of 0.05 was used here. After normalization, the MLR equation can be expressed as Eq 1.

$${\hat{Z}}_{sum}=\sum B_{k}FS_{k}$$ 1

Where ${\hat{Z}}_{sum}$ is the standard normalized deviate of the sum of the chemical concentrations, *B*_*k*_ represents the regression coefficients, and *FS*_*k*_ are factor scores calculated by the PCA analysis. The mean percentage contribution can be calculated by *B*_*k*_/∑ *B*_*k*_, and the contribution of each source *k* was estimated as Eq 2.

$$\text{Contribution of source}\;k\;\left(\text{ng}/\text{L}\right)=mean\left[Z_{sum}\right]\times \left(B_{k}/\sum B_{k}\right)+B_{k}\sigma FS_{k}$$ 2

More information of PCA-MLR in environmental studies can be found in the literatures [30,31].

## Results and Discussion

### Occurrence of ECs

**Fig 2:**
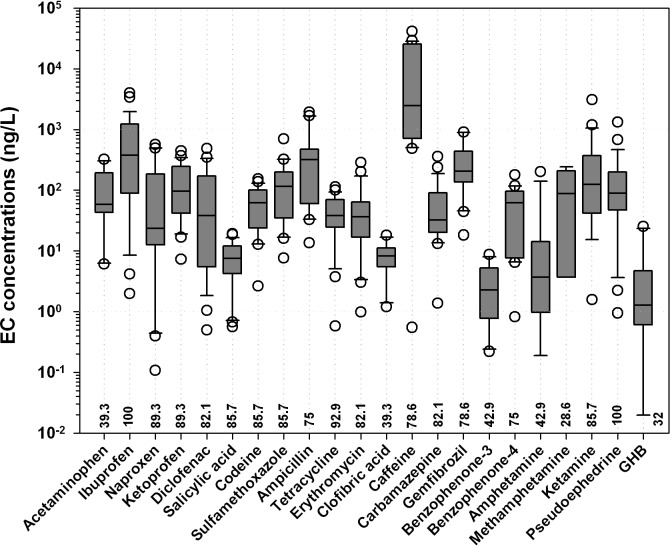
Concentration ranges of emerging contaminants in the water systems in two sampling campaigns. The solid bar makes the median. The box denotes the 0.25 and 0.75 percentiles. The whiskers mark the last value within a range of 1.5 times the 0.25 and 0.75 percentiles. Outliers are marked by dots. The values at the x-axis show the detection frequency.

The results can be illustrated better by dividing the 28 ECs into 6 groups based on their general uses and/or origins: non-steroidal anti-inflammatory drugs (NSAIDs), illicit drugs, personal care products, antibiotics, caffeine, and other pharmaceuticals (clofibric acid, gemfibrozil, and carbamazepine). The high overall frequency of detection for ECs is likely influenced by the study design, which places a focus on sampling sites generally considered susceptible to contamination (i.e., downstream of intense population, levels of urbanization, and livestock production). A large proportion of the ECs (22 out of 28) are detected at least once (Fig 2). Among the 22 detected ECs, ibuprofen and pseudoephedrine were detected in 100% of samples (S2 Table). Measured concentrations are generally low (median detectable concentrations generally < 1000 ng/L); the exception is caffeine (2792 ng/L), with a maximum concentration of 41,200 ng/L. Caffeine shows the highest concentration, with a high frequency of detection, which is not surprising, given its prevalence in beverages, foods, and pharmaceuticals [32]. Ibuprofen is detected in all surface water samples at concentrations ranging from 1.9 to 4000 ng/L. This observation is similar to findings reported in previous research [33,34] and might be explained by the fact that ibuprofen is a commonly used antiphlogistic drug, with widespread use in the treatment of symptoms of colds, aches, and pains, and for treatment of arthritic conditions [25].

Concentration ranges of ECs found in this study are listed in Table 3, which also summarizes those reported worldwide in the literatures [27,35–53]. Concentrations detected in this study are generally comparable to those from rivers in Japan, Korea, China, India, UK, and Spain, but slightly lower than those reported in the US (Table 3). Observed differences between data from Taiwan and data from other countries can be either site specific or due to general differences in prescribing patterns among countries. In addition, the possible explanation for this pattern in Taiwan may be due to the misuse of Taiwan’s National Health Insurance (NHI) program. NHI program was launched in 1995, and the NHI coverage rate has now reached 99.6%. This program provides universal health coverage and its benefit package is comprehensive; all necessary medical services are covered. The package covers inpatient and outpatient services, dental work, traditional Chinese medicine, and provides access to nearly 20,000 prescription drugs [54]. Therefore, misuse of this system may lead to large amounts of unnecessary pharmaceutical distribution, increasing direct disposal of unused medicine, releasing it into the aquatic environment.

**Table 3:** Comparison of EC concentrations in surface waters (ng/L) in the present study with those reported worldwide.

Compounds	Asia								Europe		America
	Taiwan				Japan	Korea	China	India	UK	Spain	USA	
	Gaoping River	Love River	Houjin River	Dianbao River									
**Acetaminophen**	BDL-323	BDL -185	BDL -210	BDL	BDL -263	BDL -73			BDL -2382^[39]^	BDL -872^[41]^	BDL -10000^[45]^
**Diclofenac**	BDL -16	BDL -350	38–329	33–44	BDL -220	0.87–30^[36]^	150^[27]^	BDL-26	BDL -261^[39]^	BDL -148^[41]^	BDL -177.1^[49]^
**Ibuprofen**	4.2–313	348–4000	416–2606	102–816	BDL -77	1.2–51^[36]^	685^[27]^	BDL-27	BDL -100^[39]^	BDL -541^[41]^	BDL -1000^[45]^
**Ketoprofen**	BDL -128	17–128	89–341	290–371	BDL -820		BDL-31^[50]^	BDL-16	BDL -14^[39]^	BDL -1060^[41]^	
**Naproxen**	BDL -19	11–210	38–410	BDL -22		5.3–100^[36]^	125^[27]^	BDL-1.7	BDL -146^[39]^	BDL -109^[41]^	BDL -135.2^[46]^
**Salicylic acid**	7.9–19	BDL -7.8	BDL -5.2	BDL -8.4			14736^[27]^		BDL -302^[39]^		
**Codeine**	BDL -99	13–108	64–137	42–100					BDL -815^[39]^	BDL -52^[42]^	BDL -1000^[45]^
**Sulfamethoxazole**	BDL -322	16–324	110–455	53–126	BDL -160	BDL -36^[37]^	BDL -940^[38]^		BDL -4^[39]^		BDL -520^[45]^
**Ampicillin**	BDL -1684	BDL -428	BDL -610	212–336							
**Tetracycline**	BDL -72	12–112	27–74	BDL -45			BDL-320^[52]^				BDL -110^[45]^
**Erythromycin-H**_**2**_ **O**	BDL -20	4.0–126	34–243	26–54		BDL -4.8^[37]^	BDL -121^[38]^		BDL -351^[39]^	BDL -4d2^[41]^	BDL -1700^[45]^
**Clofibric acid**	BDL	BDL -11	BDL -11	BDL -18	BDL -110		18.3^[27]^		BDL -164^[39]^	BDL -6.1^[41]^	3.2–26.7^[46]^
**Gemfibrozil**	BDL -61	BDL -904	199–605	BDL -238		0.25–13^[36]^	31.2^[27]^			BDL -212^[41]^	BDL -790^[45]^
**Carbamazepine**	BDL -14	22–359	45–119	29–67	BDL -86	8.4–68^[36]^	43.1^[27]^	BDL-5.4	BDL -684^[39]^	BDL -54^[41]^	42.9–113.7^[46]^
**Caffeine**	BDL -1016	792–41200	2792–26800	BDL -728	BDL -3500	38–250^[36]^			437^[40]^		BDL -6000^[45]^
**Benzophenone-3**	BDL	BDL -6.4	BDL -5.2	BDL					BDL -44^[39]^	BDL-295^[51]^	
**Benzophenone-4**	BDL -7.8	33–180	7.2–90	BDL -41					BDL -371^[39]^		
**Amphetamine**	BDL	BDL -202	3.7–47	BDL					BDL -21^[39]^	BDL -3.4^[42]^	BDL ^[48]^
									BDL -4.3^[40]^	1.6–11.8^[43]^	
**Methamphetamine**	BDL	BDL -237	BDL -122	BDL					BDL ^[40]^	BDL -0.7^[44]^	BDL -570^[47]^
										0.3–0.7^[43]^	BDL -62.6^[48]^
**Cocaine**	BDL	BDL	BDL	BDL					14^[40]^	BDL -11.6^[43]^	
										BDL -59.2^[44]^	
**Heroin**	BDL	BDL	BDL	BDL					BDL ^[40]^	BDL ^[42–44]^	
**Ketamine**	BDL -77	180–3084	BDL -195	BDL -125					51^[40]^	BDL -415^[42]^	
**Pseudoephedrine**	BDL -176	46–680	38–821	58–112					BDL -16.5^[40]^	0.7–145^[43]^	BDL -3300^[47]^
**MDMA**	BDL	BDL	BDL	BDL					BDL -24.8^[40]^	BDL -3.4^[43]^	BDL -96^[47]^
										BDL -11.8^[44]^	
**GHB**	BDL -25	BDL -4.2	BDL	BDL							
**References**	This study				^[35]^	^[36,37]^	^[27,38,50,52]^	^[53]^	^[39,40]^	^[41–44,51]^	^[45–49]^

BDL: Below detection limit.

### Patterns and signatures

**Fig 3:**
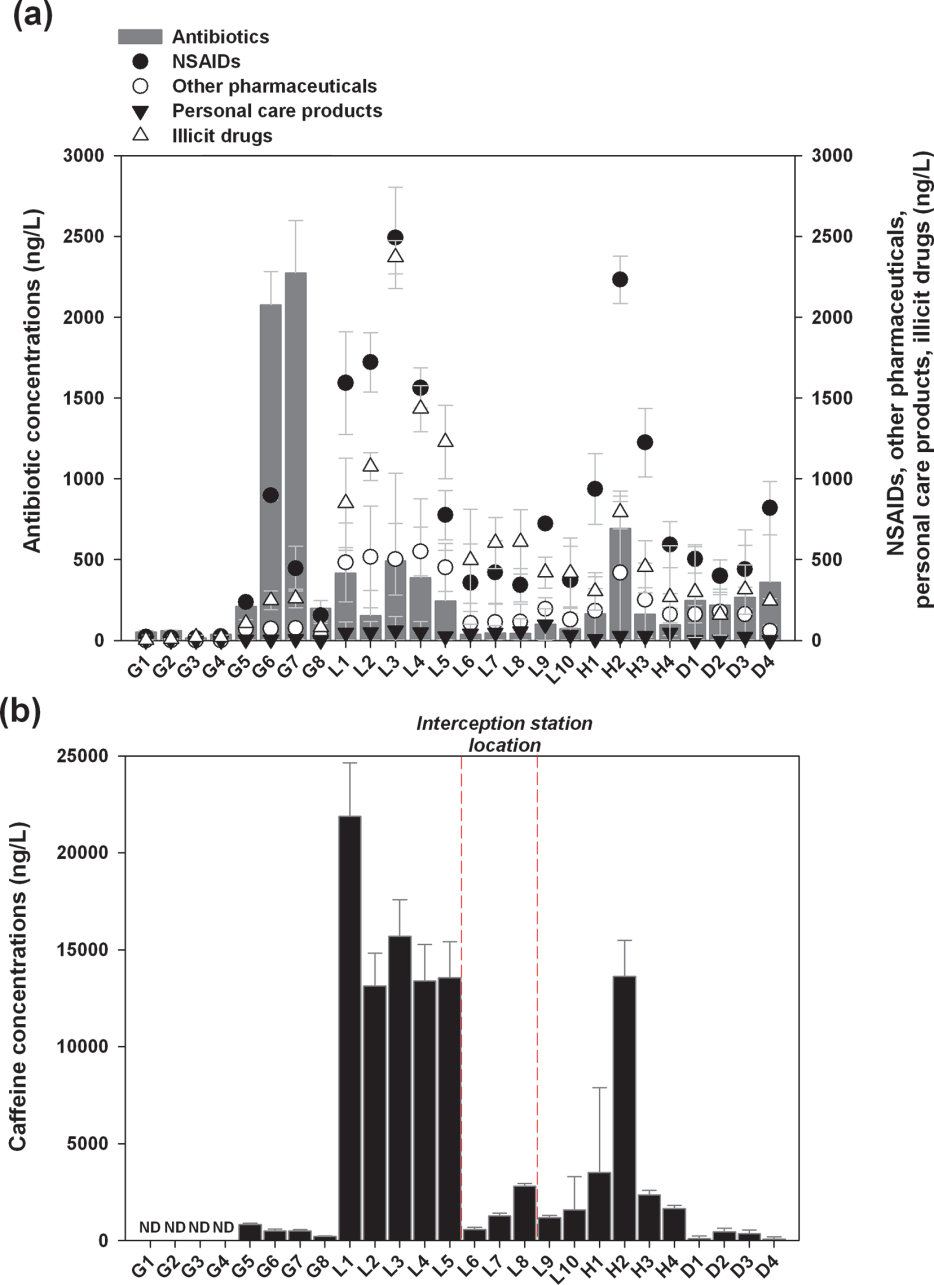
Distribution of (a) antibiotics, NSAIDs, other pharmaceuticals (clofibric acid, carbamazepine, and gemfibrozil), personal care products, illicit drugs, and (b) caffeine in all samples of the water systems.

Gaoping River is a characteristic mountain river, with a slender and sharp upstream basin. Most inhabitants (97.4%) are located in downstream areas [55]. Therefore, only scarce EC concentrations could be found at the stations G1-G4, reflecting background levels in the rural area (Fig 3). Ampicillin shows the highest concentrations of antibiotics (1920 ng/L) in Gaoping River. Animal husbandry, such as pig farming, and inappropriate disposal of manure into watercourses might explain these high antibiotic concentrations. It is estimated that there are approximately 1.9 million pigs in the drainage area of Gaoping River, approximately 30% of the entire pig production of Taiwan [56]. Thus, it is expected that there is a pronounced signal from animal husbandry. On the other hand, Ning et al. [57] find that livestock such as pig farming can be a potential threat for the water resources due to inappropriate disposal of manure into watercourses in the catchments of Gaoping River. This may represent a critical issue, as downstream waters are an important drinking water source for Kaohsiung city.

Relatively high EC concentrations were observed in upstream area of Love River. This may be so because two of the largest hospitals in Kaohsiung are located along Love River (Fig 1). Caffeine, NSAIDs, and illicit drugs have relatively high concentrations and frequencies of detection in Love River. It may reflect that cumulative contributions from domestic impact. As part of water quality management of Love River, two river interception stations were installed to collect and redirect river water for ocean outfall disposal. Hence, the downstream river waters are mainly composed of rainwater and tidal water from estuarine regions, where EC concentrations are relatively low. Higher concentrations found in Houjin River than in Dianbao River may be explained by the fact that Houjin River serves 4 times greater population in its catchment area than Dianbao River [55]. In addition, to a certain extent, Dianbao River demonstrates a similar compositional pattern with Gaoping River. The elevated concentration of antibiotics in Dianbao River may also be attributed to antibiotics use in the nearby animal husbandry area.

**Fig 4:**
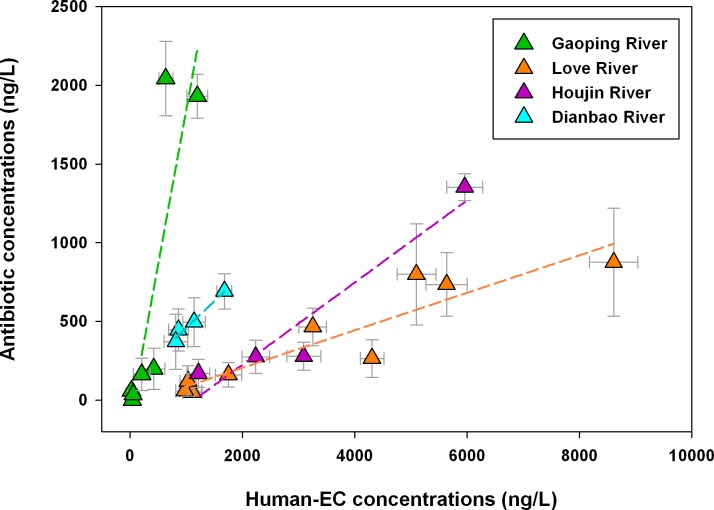
Human-EC concentrations versus antibiotic concentrations in two sampling campaigns in different water systems. Human-EC concentrations include the concentrations of NSAIDs, other pharmaceuticals (clofibric acid, carbamazepine, and gemfibrozil), personal care products, and illicit drugs. Antibiotic concentrations are the sum of sulfamethoxazole, ampicillin, tetracycline, and erythromycin-H2O concentrations.

The signatures among various rivers could be demonstrated in the plot of EC concentrations for Human-ECs (human-use drugs, including NSAIDs, clofibric acid, carbamazepine, gemfibrozil, personal care products, and illicit drugs) and antibiotic concentrations (Fig 4). A distinct skewness between human-ECs and antibiotics is found in Gaoping River and Love River. Stations in Love River and Houjin River both contained much higher concentrations of Human-ECs than antibiotics, suggesting the dominant domestic impact. On the contrary, stations in Gaoping River only have elevated levels of antibiotics, indicating an observable impact from antibiotics application on animal husbandry. In addition, a much lower concentration is observed at stations in the upstream Gaoping River (G1-G4), reflecting a signature of rural area. The results are also in agreement with the discussion mentioned above.

### Source contribution

To further identify the source contribution based on the profiles of ECs, we performed for all samples principal component analysis followed by multiple linear regression (PCA-MLR) and hierarchical cluster analysis (HCA). Concentrations below the LOQs were recorded as half of the LOQ values in the datasheet. The compounds used for multivariate analysis are shown in Table 4, and chemicals without detection or with low detection frequency were not included. PCA of the data sets in this study evolved three principal components (PCs) with eigenvalue >1. These 3 PCs were identified after varimax rotation, which accounted for 30%, 18%, and 17% of the total variance, respectively. It may be due to the missing values and replaced by half of the LOQ values of EC contaminants giving low variation in the data. Thus, some PCs captured low variance in PCA analysis [58,59]. The first component (PC1) is highly associated with diclofenac, ibuprofen, naproxen, ketoprofen, erythromycin-H_2_O, gemfibrozil, carbamazepine, caffeine, benzophenone-3, benzophenone-4, and pseudoephedrine, which are important chemicals in the human profile. Thus, PC1 could be highly indicative of the source due to domestic sewage discharging into the environment. The second component (PC2) is characterized by high loadings of sulfamethoxazole, ampicillin, tetracycline, and erythromycin-H_2_O. Chang et al. [60] investigated overall antibiotic consumption in both humans and animals in Taiwan. Annual consumption of human-use antibiotics is estimated at 329–378 tons, while 869–1,040 tons is estimated for animal-use antibiotics. This indicates that animal-use antibiotics account for 70%-76% of the total quantity of antibiotics consumed, suggesting that consumption of antibiotics in Taiwan is mainly for animal-use. Based on this profile, antibiotics application in animal husbandry area near those sites was speculated to be the potential source. The third component (PC3) has high loadings of amphetamine, methamphetamine, ketamine, and codeine and moderate loadings of ibuprofen and pseudoephedrine. Origins of these chemicals are mainly from drug abuse although some of them may partially use for medication in hospitals. Therefore, high proportions of these drugs in PC3 could also be further clarified by drug abuse.

**Table 4:** PCA loadings of investigated ECs.

Total variance explained	PC1	PC2	PC3
	30%	18%	17%
Acetaminophen	0.202	0.106	0.070
Diclofenac	**0.847**	0.372	0.268
Ibuprofen	**0.656**	0.424	**0.575**
Naproxen	**0.922**	0.169	0.094
Ketprofen	**0.750**	0.142	-0.109
Salicylic_acid	-0.253	-0.021	-0.209
Codenie	0.306	0.275	**0.766**
Sulfamethoxazole	0.200	**0.854**	0.190
Ampicillin	-0.023	**0.744**	-0.077
Tetracycline	0.211	**0.563**	0.329
Erythromycin-H_2_O	**0.669**	**0.619**	0.168
Clofibric_acid	0.023	0.008	-0.018
Gemfibrozil	**0.917**	0.156	-0.049
Carbamazepine	**0.791**	0.331	0.439
Caffeine	**0.802**	0.057	0.379
Benzophenone-3	**0.555**	-0.128	0.255
Benzophenone-4	**0.569**	-0.026	0.315
Amphetamine	0.047	0.168	**0.932**
Methamphetamine	0.166	0.098	**0.861**
Ketamine	0.186	-0.161	**0.782**
Pseudoephedrine	**0.749**	0.033	**0.566**

Extraction Method: Principal Component Analysis.

Rotation Method: Varimax with Kaiser Normalization.

Multiple linear regression analysis with the factor score (FS_*k*_) against the standard normalized deviate of the sum concentrations of the 22 chemicals (${\hat{Z}}_{sum}$) was performed to determined the mass apportionment of the three components in all samples. The resulting equation was as follows:

$${\hat{Z}}_{sum}=0.807FS_{1}+0.133FS_{2}+0.430FS_{3}\left(R^{2}=0.963\right)$$ 3

By expanding ${\hat{Z}}_{sum}$ and rearranging terms, the MLR equation becomes:

$$Z_{sum}=0.807\sigma FS_{1}+0.133\sigma FS_{2}+0.430\sigma FS_{3}+mean\left[Z_{sum}\right]$$ 4

**Fig 5:**
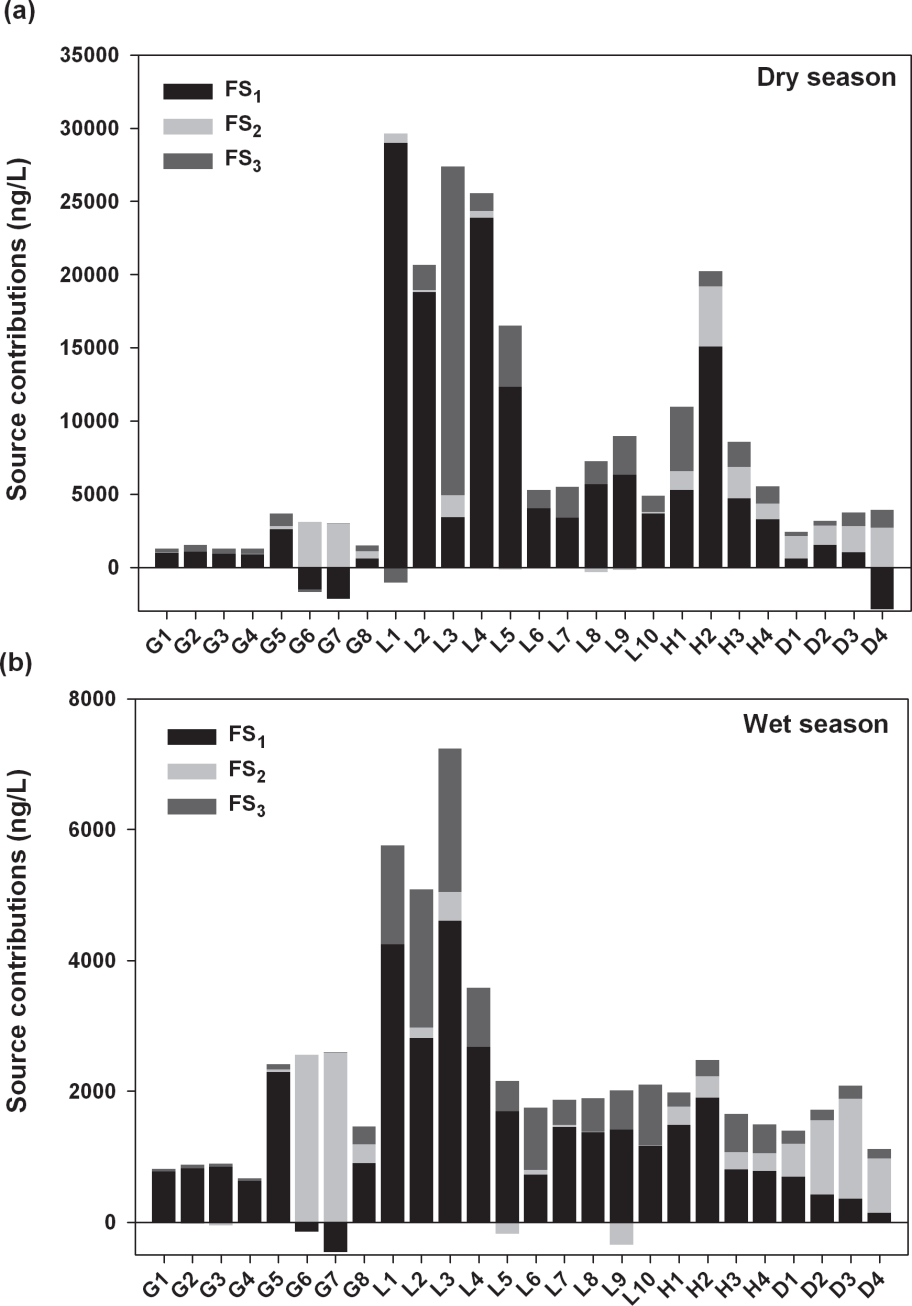
Source contributions based on principal component analysis with multiple linear regression (PCA-MLR). FS_1_: domestic impact; FS_2_: antibiotics application; FS_3_: drug abuse.

Where *σ* was 7389 ng/L; and *mean*[*Z*_*sum*_] was 5926 ng/L. Thus the mean percentage contribution (*B*_*k*_/∑ *B*_*k*_) was 58.9% for domestic impact (FS_1_), 9.7% for antibiotics application (FS_2_), and 31.4% for drug abuse (FS_3_). Fig 5 shows the estimated contributions for each source in all samples in two sampling campaigns. The positive contributions explain the variations of the source contributions in all rivers, and the negative contributions indicate the outcome of improper variable scaling inherent in PCA methods as described previously [31]. The PCA-MLR analysis showed that contributions due to antibiotics application (FS_2_) were relatively low except for samples collected near animal husbandry area (Stations G6, G7, D2, D3, and D4); these data point to antibiotics application on animal husbandry as a significant source of antibiotics contamination. The contribution levels in Love River and Houjin River were high and showed substantial domestic impact (FS_1_). The source tentatively attributed to drug abuse (FS_3_) was a contributor to most Love River samples, particularly those sites in the upstream. S1 Fig showed the relative percentage of source contribution at each sampling site. Relatively high percentage of FS_2_ was observed in Gaoping River (1.3–65% in dry season and 2.0–94% in wet season) and Dianbao River (40–63% in dry season and 37–74% in wet season), while high percentage of FS_1_ and FS_3_ were found in Love River (12–94% and 3.4–82% in dry season; 42–78% and 20–55% in wet season). These results may be consistent with land-use structure: Love River and Houjin River mainly flow through the residential areas of Kaohsiung City. Therefore, significant source contributions from domestic impact and drug abuse could be found in both two sampling campaigns for Love River and Houjin River.

**Fig 6:**
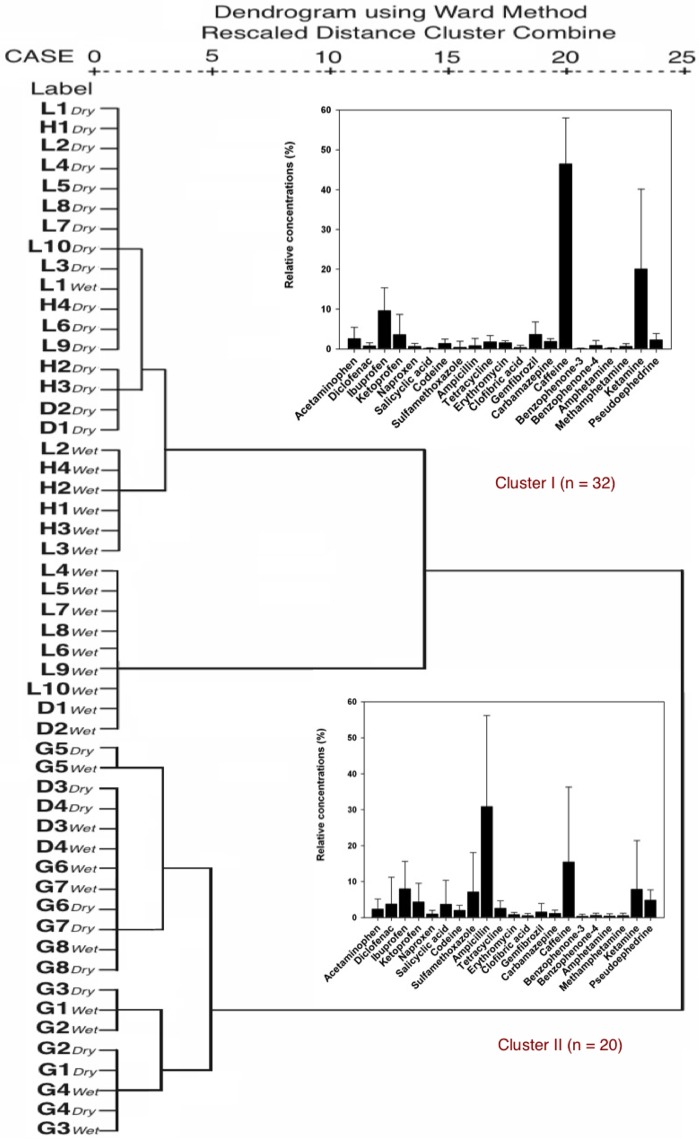
Hierarchical cluster analysis (HCA) of the water systems in two sampling campaigns, and the compositional patterns of ECs in representative clusters. The error bars represent one standard deviation of the concentrations of each compound of the relevant cluster.

The dendrogram of sampling points in two sampling campaigns obtained by HCA is shown in Fig 6. Two well-differentiated clusters were observed: (I) a cluster characterized by high compositional fractions of caffeine; and (II) a cluster characterized by high compositional fractions of ampicillin. Cluster I is the largest, formed by all stations in Love River and Houjin River and station D1 and D2. These results indicate that the signature in this cluster bears mainly domestic impacts. Cluster II comprises stations in Gaoping River and Dianbao River (G1-G8, D3, and D4). This cluster contained stations (G1-G4) with the lowest concentration of ECs, and stations characterized by high-level antibiotics. These results indicate that the signatures of cluster II were mainly derived from rural and animal husbandry contributions. These findings gave similar results and provided further evidence to source contributions.

### Environmental risk characterization

Environmental risks to aquatic organisms are assessed for a worst case scenario in southern Taiwan based on the RQ calculated using maximum MECs and PNECs (Table 5). Overall, ampicillin has the highest RQ, and the values in Gaoping River, Love River, Houjin River, and Dianbao River are 22.45, 5.71, 8.13, and 4.48, respectively. Both RQ values for ampicillin and codeine in the four rivers exceed 1.0, indicating their potential risk to aquatic organisms. Ibuprofen and diclofenac may pose a high risk to aquatic organisms in Love River and Houjin River. Similar results for these ECs with high risk are also found in surface waters worldwide. Hernando et al. [29] predict high risk levels based on the RQ values of ibuprofen, diclofenac, ketoprofen, gemfibrozil, erythromycin-H_2_O, clofibric acid, and carbamazepine in surface water and STP effluent in Europe. RQ values greater than 1.0 have been reported for ibuprofen in the Danish aquatic environment and in Spanish sewage effluent [61,62], as well as for diclofenac in a Norwegian river [63], Australian sewage effluent [64], and in the Pearl River, China [27]. In summary, risk assessment in the present study shows that ibuprofen, diclofenac, and codeine are the three NSAIDs with high ecological risk, whereas ampicillin and erythromycin-H_2_O are the two antibiotics with high ecological risk. Although direct acute ecological effects have not been reported in the aquatic environment, and the PNEC values were not derived for the most sensitive species in this study area, precautionary measures should be taken to reduce risks to aquatic organisms due to potential subtle chronic changes caused by ECs in southern Taiwan.

**Table 5:** Maximum measured environmental concentrations (MECs), predicted no effect concentrations (PNECs), and risk quotients (RQs) of each EC.

Compounds	Maximum MEC (ng/L)					RQs (Maximum MEC/PNEC)			
	Gaoping River	Love River	Houjin River	Dianbao River	PNEC (ng/L)	Gaoping River	Love River	Houjin River	Dianbao River
Acetaminophen	323	185	210	BDL	9200	0.035	0.02	0.023	0
Diclofenac	16	350	329	44	100	0.16	**3.5**	**3.29**	0.44
Ibuprofen	313	4000	2606	816	2000	0.157	**2**	**1.303**	0.408
Ketoprofen	128	128	341	371	15600	0.008	0.008	0.219	0.238
Naproxen	19	210	410	22	20000	0.001	0.011	0.021	0.001
Salicylic acid	19	7.8	5.2	8.4	60000	0.0003	0.0001	0.0001	0.0001
Codeine	99	108	137	100	60	**1.65**	**1.8**	**2.28**	**1.67**
Sulfamethoxazole	322	324	455	126	20000	0.0161	0.0162	0.0228	0.0063
Ampicillin	1684	428	610	336	75	**22.5**	**5.71**	**8.13**	**4.48**
Tetracycline	72	112	74	45	90	0.8	**1.24**	0.82	0.5
Erythromycin-H_2_O	20	126	243	54	40	0.5	**3.15**	**6.08**	**1.35**
Clofibric acid	BDL	11	11	18	1000	0	0.011	0.011	0.018
Gemfibrozil	61	904	605	238	1000	0.061	0.904	0.605	0.238
Carbamazepine	14	359	119	67	2500	0.0056	0.1436	0.0476	0.0268
Caffeine	1016	41200	26800	728	10^7^	0.0001	0.0041	0.0027	0.0001
Benzophenone-3	BDL	6.4	5.2	BDL	3900	0	0.0016	0.0013	0
Benzophenone-4	7.8	180	90	41	4897	0.0015	0.0368	0.0184	0.0083

BDL: Below detection limit.

### Limitation, advantage and application

One important limitation of developing this pharmaco-signature is the selection of the most representative and indicative target compounds. For example, several EC compounds have different applications and may be used for both human and veterinary treatment, and therefore, no distinct pattern could be observed. The use of ECs may also vary among countries. Thus, the greater difficulty lies in proper source identification. It is important for researchers that should strive to include key EC source markers that will improve the ability to identify the pharmaco-signature from this concept.

Despite the limitations, this methodology revealed several advantages. In the step-by-step approach, the first step is determining concentration distribution in terms of individual ECs, species groups, and percentages to summed EC concentrations, which can then be used to identify abundant chemicals and to clarify patterns and signatures. The second step is implementing PCA-MLR method to resolve predominant factors and source contributions. The third step is using HCA method to obtain differentiated clusters. PCA-MLR or HCA method alone cannot clearly characterize EC sources. Performing both of these methods enable to confirm and support each other and can clarify the potential source contributions.

In this study, PCA-MLR and HCA analysis were used to identify source contribution and to clarify patterns and signatures by comparing two sampling seasons despite those sampling campaigns were 3 years apart. The study showed that both PCA-MLR and HCA analysis gave similar results of pharmaco-signature in those sampling campaigns, indicating the universally coincident land-use in multi-scape water systems. Therefore, these results can strengthen the belief in the validity of these multivariate statistical analysis approaches in our study area in clarifying the potential source contributions.

The results of this concept have much broader implications for discerning source contributions. Where appropriate contaminant data are available, use of the developed methodology, with some additional perspectives geographical/hydrological characteristics of the study area, water quality parameter (e.g. BOD, TOC, *E*. *coli*), and chemical markers (e.g. pesticides, VOCs), makes it more applicable for environmental studies to further resolve potential source contributions and identification.

## Supporting Information

S1 Fig Relative percentage of source contributions based on principal component analysis with multiple linear regression (PCA-MLR). FS_1_: domestic impact; FS_2_: antibiotics application; FS_3_: drug abuse.
(TIFF)

S1 Table CAS number, formula, molecular weight, logK_ow_, logK_oc_, melting point, vapor pressure, and solubility of the selected ECs. (DOCX)

S2 Table The Rank of ECs according to the frequency of detection in the study area. (DOCX)

S1 Text Materials and Methods. Detailed descriptions of chemicals and standards, LC-MS/MS analysis, and environmental risk assessment in this study were provided in the S1 Text.
(DOCX)
