# Correction: Source Apportionment and Risk Assessment of Emerging Contaminants: An Approach of Pharmaco-Signature in Water Systems

**DOI:** 10.1371/journal.pone.0129410

**Published:** 2015-05-26

**Authors:** The PLOS ONE Staff

Hyphens were incorrectly omitted from the first three authors’ first names. The correct names are: Jheng-Jie Jiang, Chon-Lin Lee, and Meng-Der Fang. The correct citation is: Jiang J-J, Lee C-L, Fang M-D, Boyd KG, Gibb SW (2015) Source Apportionment and Risk Assessment of Emerging Contaminants: An Approach of Pharmaco-Signature in Water Systems. PLoS ONE 10(4): e0122813. doi:10.1371/journal.pone.0122813. The publisher apologizes for these errors.
