# Supplementary figures and images for: Source Apportionment and Risk Assessment of Emerging Contaminants: An Approach of Pharmaco-Signature in Water Systems
Source: PLoS One. 2015 Apr 15;10(4):e0122813. doi: 10.1371/journal.pone.0122813 (PMC4398383; doi:10.1371/journal.pone.0122813)

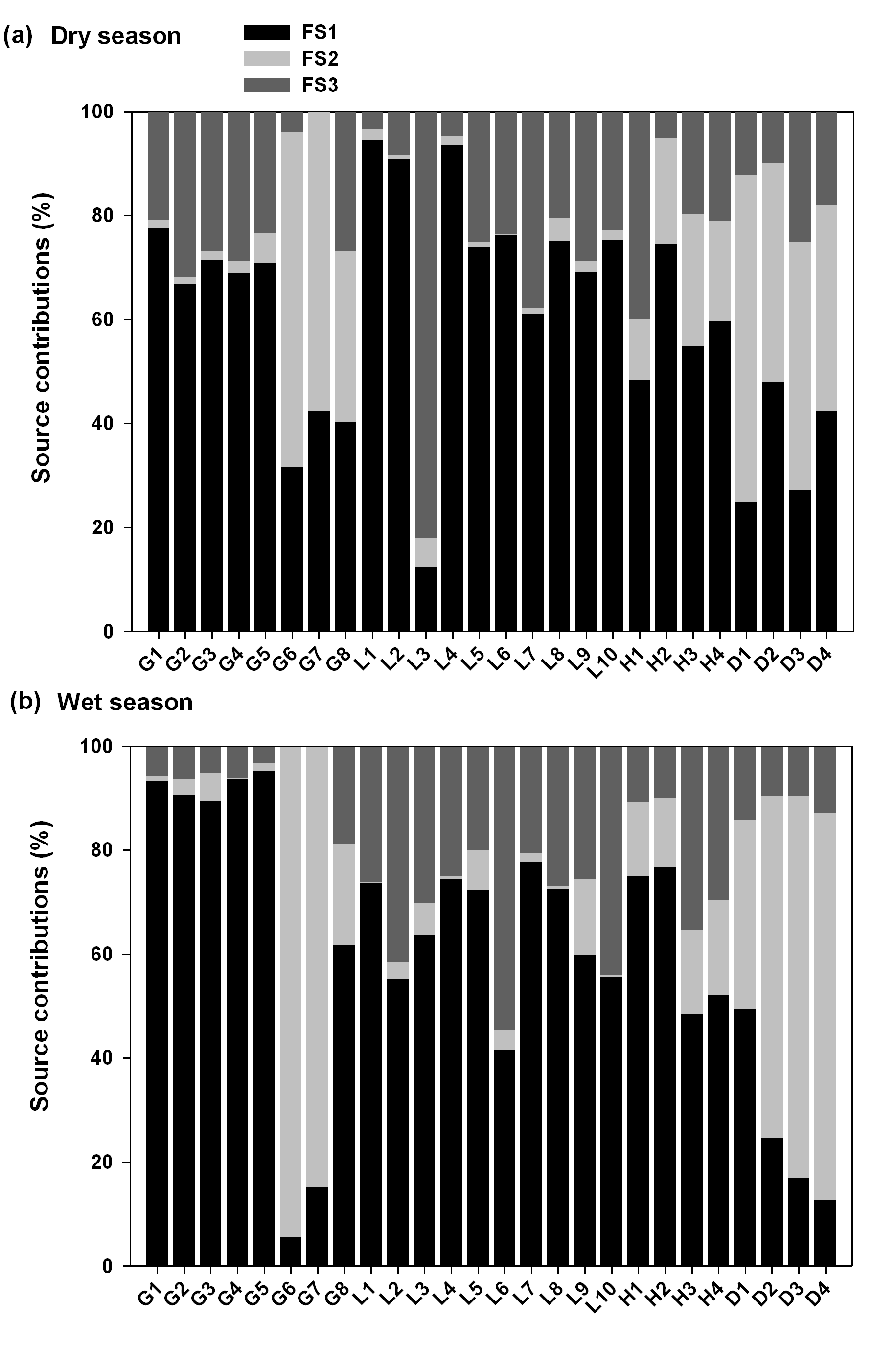

Supplement: S1 Fig — FS1: domestic impact; FS2: antibiotics application; FS3: drug abuse. (TIFF) [file pone.0122813.s001.tiff]
