# Supplementary material for: Source Apportionment and Risk Assessment of Emerging Contaminants: An Approach of Pharmaco-Signature in Water Systems
Source: PLoS One. 2015 Apr 15;10(4):e0122813. doi: 10.1371/journal.pone.0122813 (PMC4398383; doi:10.1371/journal.pone.0122813)
Supplement: S1 Table — (DOCX) [file pone.0122813.s002.docx]

Table S1. CAS number, formula, molecular weight, logK_ow_, logK_oc_, melting point, vapor pressure, and solubility of the selected ECs.

| Sample Name | CAS No. | Formula | Molecular  weight | logK_ow_ | logK_oc_ | Melting point  (℃) | Vapor pressure  (mmHg 25) | Solubility (g/L) |
| --- | --- | --- | --- | --- | --- | --- | --- | --- |
| **NSAIDs** | | | | | | | | |
| Acetaminophen | 103-90-2 | C_8_ H_9_ N O_2_ | 151.17 | 0.46 | 1.654 | 170 | 1.94x10^-6^ | 14 |
| Diclofenac | 15307-79-6 | C_14_ H_10_ Cl_2_ N O_2_ Na | 318.14 | 0.7 | 2.661 | 284 | 3.13x10^-14^ | 2.43^b^ |
| Ibuprofen | 15687-27-1 | C_13_ H_18_ O_2_ | 206.28 | 3.97 | 2.626 | 76 | 1.86x10^-4^ | 0.021 |
| Ketoprofen | 22071-15-4 | C_16_ H_14_ O_3_ | 254.29 | 3.12 | 2.586 | 94 | 1.46x10^-6^ | 0.051 |
| Naproxen | 22204-53-1 | C_14_ H_14_ O_3_ | 230.27 | 3.18 | 2.525 | 153 | 1.27x10^-6^ | 0.016 |
| Salicylic acid | 69-72-7 | C_7_ H_6_ O_3_ | 138.12 | 2.26 | 1.336 | 158 | 3.19x10^-5^ | 2.24 |
| Codeine | 76-57-3 | C_18_ H_21_ N O_3_ | 299.37 | 1.19 | 2.845 | 280 | 1.91x10^-10^ | 9 |
| **Antibiotics** | | | | | | | | |
| Sulfamethoxazole | 723-46-6 | C_10_ H_11_ N_3_ O_3_ S | 253.28 | 0.89 | 2.412 | 167 | 1.30x10^-7^ | 0.61 |
| Ampicillin | 69-53-4 | C_16_ H_19_ N_3_ O_4_ S | 349.41 | 1.35 | 1.926 | 198 | 2.84x10^-13^ | 10.1 |
| Tetracycline | 60-54-8 | C_22_ H_24_ N_2_ O_8_ | 444.44 | -1.3 | 1.644 | 178 | 2.08x10^-21^ | 0.23 |
| Erythromycin-H_2_O | 114-07-8 | C_37_ H_67_ N O_13_ | 733.95 | 3.06 | 2.754 | 191 | 2.12x10^-25^ | 5.17x10^-4b^ |
| **Lipid regulator** | | | | | | | | |
| Clofibric acid | 882-09-7 | C_10_ H_11_ Cl O_3_ | 214.65 | 2.57 | 1.64 | 118-119 | 7.54x10^-5^ | 0.58^b^ |
| Gemfibrozil | 25812-30-0 | C_15_ H_22_ O_3_ | 250.34 | 4.77^a^ | 2.636 | 62 | 3.05x10^-5^ | 4.96x10^-3b^ |
| **Antiepileptic drugs** | | | | | | | | |
| Carbamazepine | 298-46-4 | C_15_ H_12_ N_2_ O | 236.28 | 2.45 | 3.123 | 190.2 | 8.80x10^-8^ | 0.112 |
| **Psychostimulants** | | | | | | | | |
| Caffeine | 58-08-2 | C_8_ H_10_ N_4_ O_2_ | 194.19 | -0.07 | 1 | 238 | 7.33x10^-9^ | 21.6 |
| **Ulcer healing** | | | | | | | | |
| Omeprazole | 73590-58-6 | C_17_ H_19_ N_3_ O_3_ S | 345.42 | 2.23 | 3.163 | 156 | 1.16x10^-11^ | 0.082^b^ |
| **Sunscreen agents** | | | | | | | | |
| Benzophenone-3 | 131-57-7 | C_14_ H_12_ O_3_ | 228.25 | 3.79 | 2.98 | 65.5 | 6.62x10^-6^ | 0.069 |
| Benzophenone-4 | 4065-45-6 | C_14_ H_12_ O_6_ S | 308.31 | 0.37^a^ | 1.825 | 145 | 1.34x10^-11^ | 250 |
| **Illicit drugs** | | | | | | | | |
| Amphetamine | 300-62-9 | C_9_ H_13_ N | 135.21 | 1.76 | 2.883 | 11.3 | 3.1x10^-1^ | 28^b^ |
| Methamphetamine | 537-46-2 | C_10_ H_15_ N | 149.24 | 2.07 | 2.951 | 172.5 | 4.48x10^-3^ | 13.3^b^ |
| Cocaine | 50-36-2 | C_17_ H_21_ NO_4_ | 303.36 | 2.3 | 2.9 | 98 | 1.29x10^-5^ | 1.8 |
| Heroin | 561-27-3 | C_21_ H_23_ NO_5_ | 369.42 | 1.58 | 3.42 | 173 | 7.59x10^-10^ | 0.6 |
| Ketamine | 6740-88-1 | C_13_ H_16_Cl NO | 237.73 | 2.18 | 3.062 | 92.5 | 5.15x10^-5^ | 200 |
| Pseudoephedrine | 90-82-4 | C_10_ H_15_ NO | 165.24 | 1.13 | 1.856 | 40 | 1.07x10^-2^ | 63.6 |
| Cannabinol | 521-35-7 | C_21_ H_26_ O_2_ | 310.44 | 7.23^a^ | 5.517 | 77 | 7.25x10^-8^ | 2.1x10^-6^ |
| Flunitrazepam (FM2) | 1622-62-4 | C_16_ H_12_ F N_3_ O_3_ | 313.29 | 2.06 | 4.075 | 166-167 | 5.96x10^-9^ | 0.073 |
| 3,4-methylenedioxy-N-methylamphetamine  (MDMA) | 42542-10-9 | C_11_ H_15_ NO_2_ | 193.25 | 2.15 | 2.357 | 150 | 2.27x10^-4^ | 7.03 |
| Gamma-hydroxybutyric acid (GHB) | 591-81-1 | C_4_ H_8_ O_3_ | 104.11 | - 0.4^a^ | 0 | 45.54 | 3.69x10^-3^ | 1x10^3^ |
| ^a^ Estimated by KOWWIN v1.68  ^b^ Water Solubility Estimate from Log Kow  Data from EPI suite U.S. Environmental Protection Agency. | | | | | | | | |
