# Supplementary material for: Source Apportionment and Risk Assessment of Emerging Contaminants: An Approach of Pharmaco-Signature in Water Systems
Source: PLoS One. 2015 Apr 15;10(4):e0122813. doi: 10.1371/journal.pone.0122813 (PMC4398383; doi:10.1371/journal.pone.0122813)
Supplement: S2 Table — (DOCX) [file pone.0122813.s003.docx]

Table S2. The Rank of ECs according to the frequency of detection in the study area.

| Rank | Chemicals | Frequency of detection (%) |
| --- | --- | --- |
|  |  |  |
| 1 | Ibuprofen | 100 |
| 1 | Pseudoephedrine | 100 |
| 3 | Tetracycline | 92.9 |
| 4 | Naproxen | 89.3 |
| 4 | Ketoprofen | 89.3 |
| 6 | Salicyclic acid | 85.7 |
| 6 | Codeine | 85.7 |
| 6 | Sulfamethoxazole | 85.7 |
| 6 | Ketamine | 85.7 |
| 10 | Diclofenac | 82.1 |
| 10 | Erythromycin-H_2_O | 82.1 |
| 10 | Carbamazepine | 82.1 |
| 13 | Caffeine | 78.6 |
| 13 | Gemfibrozil | 78.6 |
| 15 | Ampicillin | 75.0 |
| 15 | Benzophenone-4 | 75.0 |
| 17 | Benzophenone-3 | 42.9 |
| 17 | Amphetamine | 42.9 |
| 19 | Acetaminophen | 39.3 |
| 19 | Clofibric acid | 39.3 |
| 21 | GHB | 32.0 |
| 22 | Methamphetamine | 28.6 |
| 23 | Omprazole | 0 |
| 23 | Cocaine | 0 |
| 23 | Heroin | 0 |
| 23 | Cannabinol | 0 |
| 23 | Flunitrazepam | 0 |
| 23 | MDMA | 0 |
