# Supplementary material for: Source Apportionment and Risk Assessment of Emerging Contaminants: An Approach of Pharmaco-Signature in Water Systems
Source: PLoS One. 2015 Apr 15;10(4):e0122813. doi: 10.1371/journal.pone.0122813 (PMC4398383; doi:10.1371/journal.pone.0122813)
Supplement: S1 Text — Detailed descriptions of chemicals and standards, LC-MS/MS analysis, and environmental risk assessment in this study were provided in the S1 Text. (DOCX) [file pone.0122813.s004.docx]

**Supporting Information**

**Text S1. Materials and Methods.**

**Chemicals and standards.**

All standards were obtained from Cerilliant (Round Rock, TX, USA), except sulfamethoxazole, tetracycline, erythromycin-H_2_O, and clofibric acid, which were obtained from Sigma-Aldrich (St. Louis, MO, USA); benzophenone-3 and benzophenone-4 from Fluka (Buchs, Switzerland); and ^13^C_3_-caffeine and ^13^C_6_-ibuprofen from Cambridge Isotope Laboratories (Andover, MA, USA). Detailed physicochemical properties of the 28 selected ECs are presented in the Table S1. Most of the target ECs are commonly used for prescription medication, over-the-counter remedies, human treatment, veterinary medicine, and drugs of abuse in Taiwan. These ECs are also reported in many other locations, such as USA, Europe, Japan, Korea, and China [[1-10](#_ENREF_1)]. Methanol and acetonitrile were of HPLC grade, from Merck Co. (Darmstadt, Germany). ACS-grade formic acid and hydrochloric acid were purchased from Fluka (Buchs, Switzerland). ACS-grade ammonium acetate was purchased from Sigma-Aldrich (St. Louis, MO, USA). Disodium ethylenediamine tetra-acetate (Na_2_EDTA) was analytical grade and obtained from Mallinckrodt Baker (Phillipsburg, PA, USA). Stock standard solution (1000 mg/L) was prepared in methanol and stored in amber glass bottles at −20°C for a maximum of 15 days. Working solutions were prepared by diluting the stock standard solution in methanol.

**LC-MS/MS analysis**

Chromatography was performed using an Agilent 1200 module (Agilent Technologies, Palo Alto, CA, USA). The injection volume for PPCPs and illicit drugs was 50 and 10 μL, respectively, and the auto-sampler was operated at room temperature. Separation of PPCPs was performed on a 150 × 4.6 mm ZORBAX Eclipse XDB-C18 column with a 5 μm particle size (Agilent, Palo Alto, CA, USA) and a mobile phase consisting of 0.1% formic acid (v/v) and 5mM ammonium acetate in DI water (mobile phase A) and 0.1% formic acid (v/v) in methanol (mobile phase B) gradient. The flow rate was kept constant at 1.0 mL/min. The gradient began with 0% mobile phase B for 0.5 min, increasing to 40% from 0.5-3.0 min, to 70% from 3.0-7.5 min, to 95% from 7.5-9.0 min, and remaining at 95% until 11 min, decreasing to 0% from 11 to 12 min, and remaining at 0% thereafter. All PPCPs were eluted out of the column within 15 min. Illicit drugs were separated on a Kinetex PFP column (Phenomenex, Torrance, CA, USA, 100 × 2.1 mm, 2.6 μm) at a flow rate of 0.3 mL/min. A binary gradient program was used with 0.1% formic acid (v/v) in DI water as mobile phase A, and 0.1% formic acid (v/v) in acetonitrile as mobile phase B. The gradient started at 50% of mobile phase B for 0.1 min, linearly increased to 95% mobile phase B within 6 min, returned to the initial condition of 50% mobile phase B and remained at 50% thereafter. All target illicit drugs were eluted out of the column within 10 min.

Mass spectrometry was performed using an API 4000 triple quadrupole mass spectrometer (Applied Biosystems, Foster City, CA) with an electrospray ionization source set to operate in positive/negative mode. Ions were acquired in multiple reaction monitoring (MRM) modes with a dwell time of 200 ms and unit mass resolution on both mass analyzers. Two MRM pairs were used to identify the target compounds (Table S2). Conditions for the mass spectrometer were as follows: ion spray voltage at 5.0 kV; curtain gas, nebulizer gas, and turbo gas at 10, 60, 50 L/h respectively; heated capillary temperature at 550°C; collisionally activated dissociation at 7. After selecting the precursor ions, product ions were obtained and optimized using four key parameters: declustering potential, entrance potential, collision energy, and collision cell exit potential. Standard calibration curves were constructed by spiking with standard solutions of ECs at 0.5 - 2000 ng/L. Linearity of the calibration curves was estimated by fitting a linear mode, least-squares regression analysis in the concentration range studied. Recovery experiments were performed on DI and river waters spiked with both 500 ng/L target analytes and isotopically labelled surrogates, to estimate the precision, recovery, and accuracy of the analytical method.

**Environmental risk assessment**

The potential environmental risk of the detected ECs was assessed on the basis of the risk quotient (RQ), according to the European Technical Guidance Document (TGD) on risk assessment [[11](#_ENREF_11)]. According to the TGD, when only short-term/acute toxicity data EC_50_/LC_50_ are available, the calculation of PNEC is obtained from EC_50_/LC_50_ divided by a fixed assessment factor of 1000. Once long-term/chronic NOEC values for one, two or three trophic levels are available, the assessment factors of 100, 50 or 10, respectively, are used [[11](#_ENREF_11)]. Derivation of PNECs was based on chronic toxicity data available in the literature [[12-18](#_ENREF_12)].

**References**

1. Del Rio H, Suarez J, Puertas J, Ures P. PPCPs wet weather mobilization in a combined sewer in NW Spain. Sci Total Environ. 2013; 449: 189-198.

2. Gulkowska A, He YH, So MK, Yeung LWY, Leung HW, Giesy JP, et al. The occurrence of selected antibiotics in Hong Kong coastal waters. Mar Pollut Bull. 2007; 54: 1287-1293.

3. Kasprzyk-Hordern B, Dinsdale RM, Guwy AJ. The occurrence of pharmaceuticals, personal care products, endocrine disruptors and illicit drugs in surface water in South Wales, UK. Water Res. 2008; 42: 3498-3518.

4. Kolpin DW, Furlong ET, Meyer MT, Thurman EM, Zaugg SD, Barber LB, et al. Pharmaceuticals, hormones, and other organic wastewater contaminants in US streams, 1999-2000: A national reconnaissance. Environ Sci Technol. 2002; 36: 1202-1211.

5. Nakada N, Komori K, Suzuki Y, Konishi C, Houwa I, Tanaka H. Occurrence of 70 pharmaceutical and personal care products in Tone River basin in Japan. Water Sci Technol. 2007; 56: 133-140.

6. Thomas KV, Bijlsma L, Castiglioni S, Covaci A, Emke E, Grabic R, et al. Comparing illicit drug use in 19 European cities through sewage analysis. Sci Total Environ. 2012; 432: 432-439.

7. Walraven N, Laane R (2009) Assessing the Discharge of Pharmaceuticals Along the Dutch Coast of the North Sea. In: Whitacre DM, editor. Reviews of Environmental Contamination and Toxicology, Vol 199. New York: Springer. pp. 1-18.

8. Yang Y, Fu J, Peng H, Hou L, Liu M, Zhou JL. Occurrence and phase distribution of selected pharmaceuticals in the Yangtze Estuary and its coastal zone. J Hazard Mater. 2011; 190: 588-596.

9. Yoon Y, Ryu J, Oh J, Choi BG, Snyder SA. Occurrence of endocrine disrupting compounds, pharmaceuticals, and personal care products in the Han River (Seoul, South Korea). Sci Total Environ. 2010; 408: 636-643.

10. Zhu SC, Chen H, Li JN. Sources, distribution and potential risks of pharmaceuticals and personal care products in Qingshan Lake basin, Eastern China. Ecotox Environ Safe. 2013; 96: 154-159.

11. European-Commission. Technical Guidance Document in Support of Commission Directive 93/67/EEC on Risk Assessment for New Notified Substances and Commission Regulation (EC) No 1488/94 on Risk Assessment for Existing Substances, Part II. Brussels, Belgium. 2003.

12. Ferrari B, Mons R, Vollat B, Fraysse B, Paxeus N, Lo Giudice R, et al. Environmental risk assessment of six human pharmaceuticals: Are the current environmental risk assessment procedures sufficient for the protection of the aquatic environment? Environ Toxicol Chem. 2004; 23: 1344-1354.

13. Grung M, Kallqvist T, Sakshaug S, Skurtveit S, Thomas KV. Environmental assessment of Norwegian priority pharmaceuticals based on the EMEA guideline. Ecotox Environ Safe. 2008; 71: 328-340.

14. Hernando MD, Mezcua M, Fernandez-Alba AR, Barcelo D. Environmental risk assessment of pharmaceutical residues in wastewater effluents, surface waters and sediments. Talanta. 2006; 69: 334-342.

15. Isidori M, Lavorgna M, Nardelli A, Pascarella L, Parrella A. Toxic and genotoxic evaluation of six antibiotics on non-target organisms. Sci Total Environ. 2005; 346: 87-98.

16. Jiang JJ, Lee CL, Fang MD. Emerging organic contaminants in coastal waters: Anthropogenic impact, environmental release and ecological risk. Mar Pollut Bull. 2014; 85: 391-399.

17. Santos JL, Aparicio I, Alonso E. Occurrence and risk assessment of pharmaceutically active compounds in wastewater treatment plants. A case study: Seville city (Spain). Environ Int. 2007; 33: 596-601.

18. Ying GG, Kookana RS, Kolpin DW. Occurrence and removal of pharmaceutically active compounds in sewage treatment plants with different technologies. J Environ Monit. 2009; 11: 1498-1505.
